# Adolescent boys with autism spectrum disorder growing up: follow-up of self-reported sexual experience

**DOI:** 10.1007/s00787-016-0816-7

**Published:** 2016-01-20

**Authors:** J. Dewinter, R. Vermeiren, I. Vanwesenbeeck, Ch. Van Nieuwenhuizen

**Affiliations:** 1 Scientific Centre for Care and Welfare (Tranzo), Tilburg University, PO Box 90153 (T618), 5000 LE Tilburg, The Netherlands; 2 GGzE Centre for Child and Adolescent Psychiatry, PO Box 909 (DP1104), 5600 AX Eindhoven, The Netherlands; 3 Curium-LUMC, Leiden University Medical Centre, PO box 15, 2300 AA Leiden, The Netherlands; 4 Department of Child and Adolescent Psychiatry, VU Medical Centre Amsterdam, PO Box 303, 1115 ZG Duivendrecht, The Netherlands; 5 Interdisciplinary Social Science, Utrecht University, PO Box 80140, 3508 TC Utrecht, The Netherlands; 6 Rutgers, PO Box 9022, 3506 GA Utrecht, The Netherlands

**Keywords:** Autism spectrum disorders, Sexuality, Sexual behaviour, Adolescence

## Abstract

Systematic research on sexual development in adolescents with autism spectrum disorder (ASD) remains scant, notwithstanding the often-suggested relation between ASD, atypical, and even sexually offensive behaviours. This study compared follow-up data related to lifetime sexual experience (LTSE) in a homogeneous group of adolescent boys with ASD (*n* = 30), aged 16–20, with a matched group of boys in the general population (*n* = 60). Most boys in the ASD and control groups reported masturbation and having experienced an orgasm. The proportion of boys with ASD that had no partnered sexual experience was larger than in the control group. This difference was mostly explained by significantly fewer boys with ASD, compared with controls, who reported experience with kissing and petting; no significant differences emerged relating to more intimate partnered sexual experiences. The results suggest the existence of a subgroup of boys who have not (yet) entered the arena of partnered sexual experiences—a finding in line with research in adult samples. There were no differences relating to sexual abuse or coercion. Exploration of the partnered experiences revealed a variety of types of partners, mostly of comparable age. Several boys with ASD had not anticipated their sexual debut. Although they felt ready for it, some boys reported regret afterward. The hypothesised sexual developmental trajectories are subject to further research, but the sexual experience in this sample and the assumed developmental differences indicate the need for early, attuned, and comprehensive sexuality-related education and communication.

## Introduction

Recent research on sexuality in high-functioning adolescents and adults with autism spectrum disorder (ASD) refutes old assumptions regarding the absence of sexual interest and sexual experience in this group and counters problematising views on their sexuality [1–4]. Notwithstanding these results, other studies [5–10], clinical observations, and case studies demonstrate that healthy sexual functioning, as defined by the World Health Organisation [11], is a challenge for some adolescents and adults with ASD [12, 13]. Empirical and case studies pertaining to individuals with different levels of cognitive functioning levels describe a variety of atypical or inappropriate sexual behaviours and interests. Moreover, problems related to sexual functioning, such as showing genitals and masturbation in public, fetishism, and offending, are mentioned (for an overview see [12, 13]). Gender identity issues in individuals with ASD are also reported [14–24]. However, studies on ASD and sexuality are characterised by a variety of methodological limitations relating to the participant characteristics (different levels of cognitive functioning, different operationalisations of ASD, mixed sex groups), research methods (self vs. parent or third-party reports), absence of control groups, and limited sample sizes, which makes it difficult to generalise the results. Notwithstanding the methodological problems and inconclusive findings, the social, communicative, and repetitive behaviours and interests characterising ASD [25] could explain the emergence of some sexuality-related problems. Additionally, secondary aspects relating to ASD, such as the environment where adolescents with ASD live and are raised (e.g., single sex group homes vs. at home) [10], access to sexuality education [26], and the pharmacological treatment they receive [27], may influence sexual development. Thus, characterising the relation between sexuality development and ASD is warranted to learn what support is needed to promote sexual health in people with ASD.

Sexuality development gains momentum during adolescence after sexual maturation related to puberty. Over the past several years, a normative view on adolescent sexual development has been widely accepted [28]: the problematising views on adolescent sexual development (e.g., focus on sexually transmittable infections (STIs) and teen pregnancies) have been replaced by the idea that sexuality is a normal and positive aspect of adolescent functioning [29]. However, research on normative sexual development in adolescents with ASD is scant [11, 12]. Although longitudinal studies are important sources of information on sexual development, to date these are scarce in adolescents with ASD. All existing studies on sexuality and ASD are cross-sectional, except one [8]. Three studies controlled for the influence of age. Byers and colleagues [3] found no relation between the age of their adult participants (age range 21–73) and their sexual well-being. Similarly, in the study by Stokes and Kaur [7], no differences emerged in the relation between age (age range 10–15) and sexually inappropriate behaviours in the comparison of the ASD and control samples. In a recent study, Ginevra and colleagues [5] found more inappropriate sexual behaviours in adolescents with ASD compared with general population controls and youths with Down’s syndrome, independent of their age. In their longitudinal study, Dekker and colleagues [8] found an association between autistic traits in children at age 10–12 and parent-reported sexual problems in early adolescence (age 12–15). Although these studies offer insight into sexual functioning of adolescents and adults with ASD, they provide little information regarding sexual development and sexual experience over time in adolescence. Taken together, although the number of studies on sexual development in individuals with ASD is growing, and notwithstanding that there is increasing attention to different issues relating to sexual well-being, several questions relating to sexuality development in ASD remain, e.g., regarding factors that promote healthy sexual development, or that predict sexual problems. In general, the insight into sexual development in adolescents with ASD is only emerging, and the results need further corroboration.

In 2012–2013, the self-reported lifetime sexual experience (LTSE) in a homogeneous sample of high-functioning adolescent boys with ASD was compared with a matched control group of general population boys. The LTSE in the boys with ASD did not differ from that of controls in 2012–2013 [4]. The aim of the present follow-up study was to compare the LTSE in this group of high-functioning boys with ASD to a matched control group 2 years after the initial assessment. In order to characterise the sexual development in boys with ASD, the first goal was to compare solo, partnered, and online sexual behaviours with a general population-matched control group. Second, the change in sexual experience over the past 2 years within the ASD group was inventoried. A substantial number of boys in the general population gain partnered sexual experience between age 16 and 20 [30, 31]. Finally, additional information on partnered sexual experiences of the boys with ASD was explored.

## Methods

### Participants

All eligible candidates participated in an earlier study on sexual functioning in adolescent boys with ASD (*n* = 51) [4] and agreed to be contacted again to participate in follow-up studies (*n* = 43 of the 51 original participants). Thirty boys (age *m* = 18.62, sd = 0.96, range 16.64–20.29) of the original sample agreed to participate in the present study (see Table 1).

**Table 1:** Participant characteristics

			*n* (%) (*N* = 30)	
ASD	Autistic disorder		14	(46.7 %)
	Asperger’s disorder		16	(53.3 %)
ADOS module 4	Above autism cut-off		9	(30 %)
	Above ASD cut-off		9	(30 %)
	Below cut-off		12	(40 %)
Living situation	Home		23	(76.7 %)
	Living independently		5	(16.7 %)
	Group home		1	(3.3 %)
	Residential psychiatric treatment		1	(3.3 %)
Educational level	High		11	(36.7 %)
	Low (prevocational)		19	(63.3 %)
Intelligence (full scale IQ) (*n* = 29)^a^	(*m* = 108.07, sd = 14.4, range = 76–142)			
	Very superior	130+	1	(3.4 %)
	Superior	120–129	6	(20.6 %)
	High average	110–119	6	(20.6 %)
	Average	90–109	14	(48.2 %)
	Below average	80–89	1	(3.4 %)
	Borderline	70–79	1	(3.4 %)

^a^One missing, functioning on higher prevocational level

Participants were Dutch or Belgian boys, diagnosed with Autistic disorder or Asperger’s disorder [25] by mental health professionals. All participants were high-functioning [32]; they attended regular classes or scored in the borderline-to-higher range on a standardised intelligence measure (Full scale IQ 76–142). Florid psychotic symptomatology was the only exclusion criterion. No evidence for attrition bias was found. The participating (*n* = 30) and non-participating (*n* = 21) boys had comparable ages at *t*1 (*M*_part_ = 16.6, SE = 0.16, *M*_non-part_ = 16.7, SE = 0.14, *t*(49) = 0.522 *p* = 0.604, *r* = 0.07), educational level [high vs. low (i.e., prevocational), *χ*^2^(1) = 0.612, *p* = 0.565, *V* = 0.11], and diagnosis (Autistic disorder vs. Asperger’s disorder, *χ*^2^(1) = 0.907, *p* = 0.397, *V* = 0.13). Both groups (*M*_part_ = 7.5, SE = 0.73, *M*_non-part_ = 7.8, SE = 0.83, *t*(49) = 0.276, *p* = 0.783, *r* = 0.04) had comparable autism diagnostic observation schedule (ADOS) module 4 scores. Participants (Mdn = 2, range 0–9) and non-participants (Mdn = 3, range 0–8) did not differ regarding their LTSE at the first assessment (2012–2013) (*U* = 314, *z* = −0.20, *p* = 0.988, *r* = −0.028). No differences emerged between both groups relating to specific solo (masturbation) and partnered (kissing, petting, manual, oral, penile-vaginal and anal sex) experiences on item level.

The participant group was matched to a general population control group (*m* = 18.63, *sd* = 1.09, range 16.02–20.77). Control data were selected out of a large database (data from 3926 boys and 3915 girls, living in the Netherlands, aged 12–25) collected for a nationwide study, ‘Sex under the age of 25 II’, on sexual health in adolescents in the Netherlands [30], kindly provided by the authors. Recruitment of the participants in the population study took place through schools and Municipal Administrative Systems. The control group consisted of 60 participants, matched to the participant group based on age, educational level (low/high), living area (Southern-western region of the Netherlands), and sex (male). A stratification procedure was used, and the maximal possible control group was selected. Information on intelligence scores and psychopathology in the control group was not available. The participants in the control group in the present study differ from those in Dewinter et al. (2015) [4]: no follow-up data of the controls are available.

## Materials

### Sexuality

The participants completed a shortened version of the computerised online survey developed for the ‘Sex under the age of 25 II’ study [30]. The questionnaire for the present study consisted of 105 items. The original questionnaire had 172 items: questions that did not pertain to the focus of the present study were deleted. Participants were not obliged to answer all the questions: only those items that applied to them (e.g., boys did not receive questions about pregnancy) were hierarchically selected (e.g., if a respondent confirmed having been in a relationship, other questions relating to relationships were offered). All questions were formulated in an easy and straightforward way (see “Appendix 1”) and had a closed multiple choice answer format. Specific terminology (e.g., masturbation) was explained next to each question. All questions have good face validity. One scale, LTSE, was calculated (Cronbach’s alpha based on standardised items *α* = 0.907 in the present sample) consisting of nine questions pertaining to the most common solo (masturbation) and partnered sexual experiences (French kissing (or tongue kissing), petting (referring to caressing or fondling with clothes on), masturbating another, being masturbated, passive oral sex, active oral sex, penile-vaginal sexual intercourse, and anal sex). All other comparisons were carried out on item-level. Details about the construction of the original questionnaire can be found in the study by De Graaf and colleagues [30].

### ASD

All participants had been diagnosed with Autistic disorder or Asperger’s disorder [25] before inclusion in this study. The ADOS, fluent speech module (4) [33], was used to assess ASD characteristics in the participants. The ADOS is considered to be part of the gold standard in ASD diagnostics [34]. The first author, qualified to use the ADOS for research purposes, conducted all assessments.

### Procedure

After approval of this study by the Medical Ethical Committee Brabant, Tilburg, the Netherlands (NL49082.028.14), the first author contacted all eligible participants by e-mail or phone. The participants indicated earlier whether and how they could be contacted again. All boys received extensive information (a general invitational mail containing a leaflet on the background of this study, ethical considerations, voluntariness, the possibility to withdraw, privacy issues, the way of reporting, and the possibility to gain independent advice or file complaints) regarding the study and an informed consent document they could send back to the researcher when they agreed to participate. The parents of underage boys (<age 18) also gave written consent. The boys received a link to the online questionnaire and a neutral personal user name and password. The participants could log in and out and did not have to complete the questionnaire in one sitting; they were asked to answer the questions within 2 weeks. The first author monitored whether the questionnaires were completed and sent an additional e-mail after 2 weeks to yet again invite the participants to complete the questionnaire. One boy agreed to participate but did not fill in the questionnaire and indicated that he was too busy. During data collection, the results were stored on a secured server. The boys could indicate if they wanted to complete the questionnaire on their own or in the presence of the first author. Only one boy preferred the presence of the researcher. The boys could contact the researcher in case they had questions relating to the questionnaire or the topic. All boys received a voucher (5€) after participation.

### Statistical analysis

The main goal of this study was to compare frequencies and means relating to experience with different sexual behaviours in the participant group compared with the general population control group. Chi square (*χ*^2^, 2 by 2) tests were used to compare frequencies pertaining to item-responses. Two-tailed probabilities and Cramer’s *V,* as the effect size (*V* = 0.30 medium effect size, *V* = 0.50 large effect size [35]), were reported. Fisher’s exact test was applied when the cell frequencies were expected to be below five. Mann–Whitney (*U*) was used to compare medians and distribution of the LTSE scale, given the non-normally distributed data. Exact two-tailed probabilities were reported. The reported effect size was *r* (>50 indicates a large effect). Kendall’s tau (*τ*) was applied to check the correlations between non-normally distributed data. In order to explore the change in sexual experience, the order in which sexual behaviours were acquired was studied. Mokken scale analysis was performed in R (https://www.r-project.org) to evaluate Guttman characteristics of the LTSE scale. Loevinger’s *H* was reported (maximal value *H* = 1). No statistical testing was performed on subgroups, given the small samples sizes.

A priori power analysis (*α* = 0.05, 1−*β* = 0.80) using G*power [36] demonstrated that medium to large effect sizes (*w* > 0.03) could be detected in a total sample of *n* = 88. This sample also allowed finding large effects (*d* = 0.8) when applying 2-tailed *t* tests or non-parametric alternatives. Repeated testing in the same group increases the risk for type I errors. Given the signalling function of an explorative study, we decided to keep *α* = 0.05 and to report effect-sizes. SPSS 19 was used for data analysis.

## Results

### Sexual experience in 2014: ASD vs. controls

The ASD and control group did not differ in the percentages of boys who had experience with masturbation and reported having had an orgasm (see Table 2).

**Table 2:** Lifetime sexual experience at *t*2

	Lifetime experience				Cramer’s V	ASD Mdn	Age first time			
	ASD *n* (%) (*N* = 30)	Control *n* (%) (*N* = 60)	*Χ* ^2^ (*df* = 1)	*p*			Control Mdn	*U*	*z*	*p*
Has been in love	24 (80)	57 (95)	n/a	0.055^a^	0.236	n/a	n/a	n/a	n/a	n/a
Dating	20 (66.7)	51 (85)	4.04	0.057	0.212	15	15.5	368.5	−1.839	0.066
Masturbation	29 (96.7)	54 (90)	n/a	0.417^a^	0.117	13.5	13.5	714	−0.677	0.503
Orgasm	28 (93.3)	56 (93.3)	0.00	1.00	0.000	13.5	13.5	684	−0.969	0.336
Partnered sexual experience	21 (70)	55 (91.7)	n/a	0.012	0.282	n/a	n/a	n/a	n/a	n/a
French kissing	21 (70)	53 (88.3)	4.6	0.042	0.226	13.5	13.5	485	−0.865	0.391
Petting with clothes on	21 (70)	53 (88.3)	4.6	0.042	0.226	15.5	15.5	500	−0.691	0.494
Masturbating another	16 (53.3)	45 (75)	4.29	0.055	0.219	16	15.5	349.5	−0.177	0.864
Being masturbated	17 (56.7)	43 (71.7)	2.02	0.235	0.150	16.5	15.5	365	−0.008	0.997
Oral sex^b^ (active)	15 (50)	38 (63.3)	1.469	0.261	0.128	16.5	16.5	271	−0.284	0.784
Oral sex^b^ (passive) (*n* = 21)	17 (56.7)	39 (65)	0.591	0.494	0.081	16.5	16.5	323.5	−0.147	0.886
Penile-vaginal intercourse	17 (56.7)	40 (66.7)	0.861	0.487	0.098	16.5	16.5	315	−0.450	0.659
Anal sex^b^	3 (10)	12 (20)	1.44	0.369	0.126	17.5	17.5	15.5	−0.368	0.824

^a^Fisher’s exact

^b^Question only asked in case of other partnered experience (ASD *n* = 21, contr. *n* = 55)

However, a few more participants with ASD had no partnered sexual experience compared with controls (Fisher’s exact *p* = 0.012, Cramer’s *V* = 0.282). On an item level, only significant differences existed relating to French kissing and petting. Differences pertaining to other partnered experiences were non-significant and small. Boys in both groups reported similar ages of first experience with each of the listed sexual behaviours. LTSE (9 items) proved to be a Guttman scale (scale Loevinger’s *H* = 0.949), i.e., items could be hierarchically ordered: (1) masturbating, (2) French kissing, (3) petting, (4-6) being masturbated, receiving oral sex, sexual intercourse, (7) masturbating another, (8) oral sex to another, and (9) anal sex. A participant who scored on a specific item also scored on all subsequent items. Comparison of scale scores between both groups revealed no significant difference between the ASD group (Mdn = 7, *m* = 5.2) and controls (Mdn = 8, *m* = 6.28, *U* = 716, *z* = −1.638, *p* = 0.103, *r* = −0.173). The correlation between LTSE, age (*τ* = −0.042, *p* = 0.763), and the total ADOS score (*τ* = 0.006, *p* = 0.970) was low.

The control and ASD groups did not differ regarding the proportion of boys that used the internet for sexuality-related means (see Table 3). A substantial number of the boys without partnered experience in both groups did watch porn but did not use the internet for other sexuality-related means. The boys with ASD who used the internet for other sexuality-related means (chatting about sex, sending pictures) mostly had experience with a partner.

**Table 3:** Online sexual activity

	ASD *n* (%)	Controls *n* (%)	*Χ* ^2^ (*df* = 1)	*p*	Cramer’s *V*	Partnered experience	
						Yes (*n* = 21)	No (*n* = 9)
Talking about sex	12 (40)	23 (38.3)	0.023	1.00	0.016	11	1
Flirting	13 (43)	27 (45)	0.023	1.00	0.016	12	1
Showing genitals or buttocks	3 (10)	4 (6.7)	n/a	0.682^a^	0.059	3	0
Posting images of yourself	3 (10)	2 (3.3)	n/a	0.328^a^	0.137	3	0
Sex with someone met on the internet	2 (6.7)	3 (5)	n/a	1.00^a^	0.034	2	0
Watching porn	25 (83.3)	47 (78.3)	0.313	0.781	0.059	19	6

^a^Fisher’s exact

### Sexual development in boys with ASD

The time between the first assessment [4] and the completion of the questionnaire for the present study was on average 2.02 years (sd = 0.46). Given that the LTSE was a Guttman scale, the change in LTSE between the first assessment and follow-up could be compared. The majority of the participants without partnered experience at the first assessment did not gain experience at follow-up. Table 4 shows the change in sexual experience between the first assessment in 2012–2013 (*t*1) [4] and the present. Two boys with ASD had no sexual experience at all at t1, and one of them gained experience with masturbation in 2014. Three boys who had only solo sexual experience at *t*1 reported partnered experience at follow-up, and two boys who had been kissing and petting gained more intimate sexual experience at follow-up. Only eight out of 30 boys moved to a next level of sexual experience. At follow-up, still fewer boys with ASD had experience with French kissing and petting, the most common first partnered experiences, compared to boys in the control group, indicating that they did not take the next step in gaining sexual experience. Most boys who reported partnered experience at an earlier age evolved to more intimate sexual behaviours (manual or oral stimulation, and sexual intercourse).

**Table 4:** Sexual development

*N* = 30	*t*1 (*n*)	Change between *t*1 − *t*2 (*n*)		*t*2 (*n*)
No experience (NE)	2	No change	1	1
Solo experience (SE)	11	NE *t*1 − SE *t*2	1	8
		No change	7	
Kissing & petting (KP)	5	SE *t*1 − KP *t*2	1	3
		No change	2	
Intimate sexual experience (ISE)	12	SE *t*1 − ISE *t*2	3	18
		KP *t*1 − ISE *t*2	3	
		No change	12	

### Explorative findings relating to partnered sexual experience in the boys with ASD

Two boys with ASD reported to be forced to do sexual things against their will, compared with none in the control group. Three boys indicated having sexually coerced another, as did two boys in the control group. One boy with ASD had sexual intercourse with a professional sex-worker and had been paid himself to have sexual contact. About half the boys with ASD who had experience with sexual intercourse (*n* = 17) had two or more different sex partners (*m*_ASD_ = 3.41, *m*_contr_ = 2.28). Fourteen of these boys had followed a linear developmental trajectory [37]: they had experience with less intimate behaviours (kissing, petting) at least 1 year (*m* = 2.18, sd = 1.47) before they had more intimate experiences (oral and manual stimulation of or by the partner, sexual intercourse). Eleven boys (64.7 %) used a condom when they first had sex, comparable to the rate in the control group (77.5 %). About half the boys with ASD who had experience with sexual intercourse (ASD 52.9 %, controls 80 %) indicated they liked their first time having sexual intercourse, and six (35.3 % of the experienced boys with ASD, 12.5 % of controls) of them preferred it to have happened earlier. For about half of the boys with ASD (ASD 52.9 %, controls 20 %), the timing of their first experience having sex was unexpected. One boy indicated that he did not like his first time having sexual intercourse. After their first-time sexual intercourse, 41.2 % had regrets afterwards, compared to 7.5 % of controls. Most boys had same-aged partners (ASD 58.8 %, controls 76.2 %), and some had at least two-year-older partners (ASD 17.6 % vs. controls 7.5 %). The type of relationship with the sex partners was comparable in both groups: a romantic relationship (ASD 64.7 %, controls 65 %), a summer love (ASD 11.8 %, controls 10 %), or someone else (ASD 23.5 %, controls 25 %). In this study, only one boy with ASD had sexual experience with another boy, and one other indicated interest.

## Discussion

This study helps to elucidate the sexual development of high-functioning boys with ASD compared with general population peers. The results confirm that sexuality is part of adolescent development in boys with ASD, as it is for their peers. However, some differences pertaining to sexual experiences with a partner emerged between boys with ASD and the controls.

### Common sexual experiences in boys with ASD

Almost all boys, ASD and controls, reported having masturbated and had experienced an orgasm. The majority had some partnered sexual experience; however, fewer boys with ASD had partnered sexual experience compared with boys in the general population. This difference between boys with ASD and controls relating to partnered experience was not found 2 years prior [4]. The differences pertained to the lower number of boys with ASD who gained experience with French kissing and petting. All frequencies relating to partnered sexual behaviours seemed to be lower in the ASD group, compared with the control group; however, most of these differences did not reach significance, and most effect sizes remained small. In general, these results demonstrate the normativity of sexuality in the development of boys with ASD.

### ASD and sexual development

Within the group of boys with ASD, no relation between sexual experiences and the amount or severity of ASD features could be detected. However, LTSE, or having sexual experience, offers little insight as to the quality or context of these experiences: it might be possible that ASD features have some influence on the type or duration of romantic and sexual experiences. The absence of a relationship between ASD severity and LTSE in this study probably indicates that a majority of boys with ASD are interested in sexuality and partnered experiences. However, methodological issues, such as the sensitivity of ADOS as a measure for ASD-severity (ADOS), might also explain the absence of a relation.

The exploration of the individual developmental trajectories of the boys with ASD revealed that boys with more intimate partnered experience also had experience with all less intimate behaviours and masturbation. It should be noted that, in this study, several more intimate sexual behaviours loaded on the same level, meaning that the sequence is interchangeable. These findings differ from results in the general population [37] and might be explained by the small sample size. However, also in this study, less intimate sexual behaviours preceded the more intimate ones. A linear trajectory in gaining sexual experience [37] (from less to more intimate behaviours) is common for the majority of boys in the general population as well. Gradually gaining experience offers the opportunity to develop skills and self-knowledge to interact with a partner and to refuse unwanted experiences. This study also revealed that a small group of boys with ASD seemed slower in starting to experiment with kissing and petting, the common first steps into partnered sexuality, compared with controls.

Different possible dynamics might explain why some boys with ASD did not, yet, have partnered sexual experience: individual characteristics (e.g., hesitance to approach potential partners, no interest in dating) and contextual aspects (e.g., few potential partners available) might impede the development of sexual relationships and hamper gaining partnered sexual experience. Byers and colleagues [1] examined the sexual functioning of single adults with ASD. These researchers found a group of heterosexual adult men who had no partnered experience and who had higher levels of sexual anxiety, lower arousability, less dyadic desire, and less positive sexual cognitions. Possibly, two developmental trajectories can be discerned. The first group experiments with solo sexual behaviours and starts experimenting with partners at an age comparable with typically developing boys. Their early dating experiences may contribute to the development of relationship skills and the self-confidence to approach potential partners. A second, relatively small, group exists mostly of boys who have tried masturbation and experienced orgasm, but are slower to gain partnered experience because of internal and external barriers. Although most of these boys had watched internet porn, they did not use the internet for other sexuality-related means. The absence of partnered sexual experience does not have to be problematic: our results do not offer insight into how these boys felt about this. Last, a third developmental trajectory might exist. Two boys in this study reported no experience of an orgasm and also had no partnered experience. It might be possible that a small minority exists that does not, or to a lesser degree, experience sexual arousal. Feelings of asexuality in adults with ASD have also been previously reported [10, 38–40].

### Partnered sexual experiences in boys with ASD

More than half of the boys with ASD had sexual experience with a partner. Exploration of the partnered experiences revealed that the boys’ partners were mostly same-aged peers. More than half the boys in this study, with ASD and partnered experience, had at least two different sex partners and experimented with a variety of sexual activities. Boys in both the ASD and control groups had their first sexual experiences in different types of relationships. These results suggest that the sexual experience of the boys with ASD was mostly not occurring in a one-time only situation.

Several differences emerged between the experiences of the boys with ASD and the controls that warrant further attention, although they should be interpreted with care given the small sample size. Fewer boys with ASD seemed to be able to anticipate their first sexual intercourse. This might influence their safe sex practices and the decision-making process to consent with partnered sex: they have to decide on the spot, rather unprepared. About a third of the boys with ASD indicated that they did not use a condom when they first had sex, and half of them indicated some regrets about their first experience with sexual intercourse. Notwithstanding that their sexual debut came unanticipated for a number of boys, a third of the sexually experienced boys with ASD felt ready for sex earlier on. A small number of boys confirmed having coerced another to do sexual things, or had been forced by others. Although some of these boys reported risky, and possibly harmful, behaviours, most boys reported common and responsible experiences. The small sample and the low frequencies of these behaviours prohibit drawing firm conclusions on the prevalence of offending and victimisation. However, attention to offending and victimisation is needed to intervene and offer support as soon as possible. In addition, further research on the prevalence of offending and victimisation in adolescents with ASD is needed. The findings relating to partnered experiences stress the importance of comprehensive sexuality education with attention to social, communication, personal needs and desire, and safe-sex issues.

### Strengths, limitations, and further research

This study is, to the best of our knowledge, the first to offer insight into the sexual functioning over time of a group of adolescent boys with ASD by comparing self-reported sexual behaviours with a matched control group. This study builds on the results of a first assessment of the sexual functioning in the participant group and offers more insight into sexual development. The results of this study should, however, be interpreted in light of some limitations. First, selection bias might have occurred: the boys and their parents volunteered to participate in this study. People volunteering for sexuality-related research were found to have more sexual experiences and more positive attitudes [e.g., 41, 42]. Second, the data on lifetime sexual experience did not provide insight into the frequency and quality of sexual experience, the context, or a detailed description of the experiences. Differences pertaining to these issues might differentiate boys with ASD from their general population peers. Third, the range of intellectual functioning was broad across the participant group. Given the relation between intelligence, educational level, and sexual development in this age group [e.g., 8, 30], a smaller range would have strengthened our findings. Additionally, although the age range is limited, sexual experience can rapidly change in these years. In addition, depending on their age, the boys function in differing contexts (high school, college, university, work, in institutions or at home). Therefore, research in more homogenous samples should be considered. Last, the follow-up period between both assessments was relatively short. Differences might disappear or change later on in development. The group participating in this follow-up study was small in general and too small to study possible subgroups. The developmental trajectories described previously remain highly tentative and should be tested in further research.

Extensive follow-up of a large, homogenous group of boys with ASD would, therefore, be our primary suggestion for further research. A larger sample would allow for testing and exploring the different hypothesised developmental trajectories. Additional research on factors (e.g., anxiety, self-image) that are possibly related to or predict a specific developmental trajectory is needed. The present study revealed no clear relationship between the amount of ASD characteristics and partnered experience, so other aspects (e.g., anxiety, less desire) might be at stake. Additionally, further attention to partnered experiences (preparation, evaluation, and interaction) is needed based on the exploratory findings in this study. Further attention to sexual diversity and its relation to well-being in individuals with ASD are needed. A mixed-methods approach, combining quantitative and qualitative methods, would probably offer valuable information to gain a better insight into sexual development in adolescents and adults with ASD. Because this study offers insight only into lifetime sexual experience, sexual functioning and well-being, and relationship development of boys with and without early sexual experience later in life are subject to further research. Finally, sexuality development in girls with ASD needs attention in research.

### Clinical implications

This study’s findings support the frequently mentioned need for comprehensive and ASD-friendly sexuality education [26, 44, 45], beginning at an early age. Attention to the characteristics, and to the romantic and sexual experiences of individual boys, is necessary to tailor this education to the needs of each individual. First, the explorative and sexually active boys could benefit from discussing flirting, communication with a partner, sexual practices, and safe sex. Second, for boys without partnered experience, we could discuss the presence or absence of desire to find a partner, explore issues impeding them to find a partner, and their experience of approaching potential partners. Third, for boys without, or even with atypical, sexual interest, discussion of how they experience the absence of (typical) sexual interest, while observing sexuality and relationship development in their peers, could be helpful. Specific risks related to the different sexual developmental trajectories could also be assumed, without problematising sexuality in all boys with ASD: explorative boys might be at greater risk for getting into situations they are not yet prepared for, or getting into situations where they persuade their partners and cross boundaries; boys without partnered experience might feel different or frustrated. These specific risks might, however, also be present in typically developing boys. A more individualised approach is probably beneficial for boys with ASD. We believe that promoting sexuality-related education and communication by parents [43, 46–48], professionals, and caregivers [49] could only add to the knowledge and skills to address partners and to have sexual experience in a pleasurable, consensual, and safe way.

## Conclusions

Recent studies, including the present study, demonstrate that sexuality is part of typical adolescent development in most boys with ASD, as it is for their peers. A predominant problematising view on ASD and sexuality is again refuted, although several reasons for concern emerged (e.g., boys reporting regrets after their first time having sexual intercourse). The results of this follow-up study demonstrated mostly similarities in the sexual experience of boys with ASD and their peers in the general population. The finding that fewer boys with ASD than the controls had been kissing or petting a partner could be explained by a group of boys that are, possibly, more anxious or hesitant to approach potential partners, and by boys who experience less interest in romantic relationships or sexuality. These hypothesised trajectories should be explored in further research. In the meantime, early and comprehensive sex education, and sexuality-related communication by parents and professionals surrounding boys with ASD, attuned to their sexual interest and experience, remains important to support them in dealing with and enjoying their sexuality and romantic relationships.
